# aCGH-MAS: Analysis of aCGH by means of Multiagent System

**DOI:** 10.1155/2015/194624

**Published:** 2015-03-22

**Authors:** Juan F. De Paz, Rocío Benito, Javier Bajo, Ana Eugenia Rodríguez, María Abáigar

**Affiliations:** ^1^Biomedical Research Institute of Salamanca, BISITE Research Group, University of Salamanca, Edificio I+D+i, 37008 Salamanca, Spain; ^2^IBMCC, Cancer Research Center, University of Salamanca-CSIC, 37007 Salamanca, Spain; ^3^Department of Artificial Intelligence, Technical University of Madrid, Campus de Montegancedo, s/n Boadilla del Monte, 28660 Madrid, Spain

## Abstract

There are currently different techniques, such as CGH arrays, to study genetic variations in patients. CGH arrays analyze gains and losses in different regions in the chromosome. Regions with gains or losses in pathologies are important for selecting relevant genes or CNVs (copy-number variations) associated with the variations detected within chromosomes. Information corresponding to mutations, genes, proteins, variations, CNVs, and diseases can be found in different databases and it would be of interest to incorporate information of different sources to extract relevant information. This work proposes a multiagent system to manage the information of aCGH arrays, with the aim of providing an intuitive and extensible system to analyze and interpret the results. The agent roles integrate statistical techniques to select relevant variations and visualization techniques for the interpretation of the final results and to extract relevant information from different sources of information by applying a CBR system.

## 1. Introduction

There are various techniques for performing studies on genetic variations in patients, including expression arrays [1, 2], CGH (comparative genomic hybridization) arrays [3], and studies at the genetic sequence level. CGH arrays allow comparing the DNA of a patient with a control DNA and using this information to detect mutations [4, 5] based on gains, losses, and amplifications [6]. Another kind of microarrays is the expression arrays, which determine the expression of different genes with probes. CGH are used to detect regions in the chromosomes with variations in certain pathologies. This information is taken into account for sequencing these regions through the use of expression arrays and sequencers [7]. In these studies, the users have to work with a vast amount of information, which implies the development of systems oriented to improve the analysis of the data and to automatically extract information using databases [8]. For this reason, it is necessary to identify the exact location of those interesting genes in CGH arrays before carrying out the sequencing.

There are currently various tools that provide a visual analysis of the information of aCGH. These tools typically represent the information but the interaction with the information is complex. The visual analysis is used to represent additional information about relevant regions. Some of these tools can be found in works [9–14]. A visual analysis of these data is normally performed manually [14, 15], which requires the participation of experts to select the relevant information. However, these tools lack usability and require the use of techniques that facilitate the automatic analysis and extraction of information from different sources. For this reason, it is necessary to incorporate a process that helps determine the interesting genes [16], proteins, and relationships to diseases that must be analyzed and understood in a simpler way.

The distributed analysis of CGH data is performed by different laboratory personnel, from hybridating the chips to extracting the relevant variations and information associated with the chips. This work shows a multiagent system specifically designed to analyze CGH data [17]. The functionality of the multiagent system is divided into layers and roles to carry out the analysis of CGH arrays. The analysis is usually composed of several stages. The first stage is the segmentation process [18], which implements the subsequent analysis of the data and is important to be able to represent a visualization of the data. The remaining stages depend on the analysis to be performed and include clustering, classification, visualization, or extraction of information from databases. The proposed multiagent system manages the analysis and the automatic interpretation of the data. The system can select the relevant genes and transcripts for the prior classification of pathologies. The information of the identified genes is obtained from public databases. The information management system is based on the CBR (case-based reasoning) model [19, 20] to detect the mutations, genes, proteins, and diseases. Finally, visualization assists the user in reviewing the results.

This paper is organized as follows.  Section 2 describes the state of the art in CGH arrays, Section 3 describes the proposal, and Section 4 presents the results and conclusions.

## 2. CGH Arrays

Array-based comparative genomic hybridization (aCGH) is a kind of microarray that analyzes areas of the genome to detect gains or losses. Whereas traditional high-resolution chromosome analysis detects chromosome structure alterations at a resolution of 5 megabases (Mb) or greater, aCGH detects gains or losses of DNA that cannot be seen by traditional karyotyping and may sometimes be only thousands of base pairs in size [21]. aCGH has emerged as a powerful diagnostic technique for high resolution analysis of the human genome. It is a specific, sensitive, and rapid technique that can detect genomic arrangements and copy-number changes. A variety of array CGH platforms are currently available, both commercially and in academic institutions. The choice of platform may depend on the type of data sought; however, the price, reproducibility, and standardization are crucial factors that need to be considered [22].

CGH arrays incorporate segments of DNA that are defined with genome databases. The clones are predominantly selected to target areas of the human genome that, when deleted or duplicated, are known or highly suspected to cause well-characterized genetic defects. Microarray printers attach the clones to a glass slide in an organized way to form a microarray. A typical microarray slide contains thousands of different clones representing targeted areas of the genome. Fluorescently labeled DNA from both the patient and a known normal human control are applied to the slide and compete to attach or hybridize to their corresponding DNA segments. The fluorescent signals are analyzed and, depending of the values obtained, it is possible to detect areas with unequal hybridization of a patient versus control DNA.

The first whole genome microarray contains 2,400 large-insert genomic clones, primarily bacterial artificial chromosomes (BACs). With the total human genome covering approximately 3,000 Mb, the resolution of this array is on average close to 1 Mb, about one order of magnitude higher than that obtained with classical CGH [23]. For a full coverage resolution array, approximately 30,000 BACs have been arrayed [24], increasing the resolution with another order of magnitude. However, producing such large numbers of BACs for array CGH is expensive and time-consuming and, due to the large size of the BACs, the limits of BAC array CGH resolution have been reached. These problems can be overcome when oligonucleotides are used as targets in microarray experiments. Oligonucleotides allow a sheer infinite resolution, great flexibility and are cost-effective [3]. Moreover, oligonucleotides allow for the generation of microarrays for any organism for which the genome has been sequenced. Attempts have been undertaken to increase the resolution of BAC arrays in other ways, but CGH cannot compete with the flexibility and versatility of oligonucleotides. Finally, oligonucleotide arrays are being used, designed, and accepted for expression profiling and are thus widely available [25].

In conclusion, BAC arrays (array-based comparative genomic hybridization) have proved to be successful for the detection of submicroscopic DNA copy-number variations. Technological improvements to achieve a higher resolution have resulted in the generation of additional microarray platforms encompassing larger numbers of shorter DNA targets (oligonucleotides). Currently, both types (BAC and oligo arrays) have advantages and disadvantages. The BAC clone targets have been mapped to the human reference sequence produced by the International Human Genome Sequencing Consortium, allowing easy access to information in the related genomic databases. However, BACs, which are usually 100–200 kb, may miss alterations smaller than the size of a clone but are less likely to detect alterations of unclear clinical significance. For this reason, they are unable to distinguish deletions/amplification less than approximately 85 kb.

Oligonucleotides, which are much smaller probes, usually 25–60 bp, may detect small alterations that would not be seen using a BAC microarray, but oligonucleotide arrays are more likely to detect small alterations of unclear clinical significance.

## 3. Multiagent System

**Figure 1:**
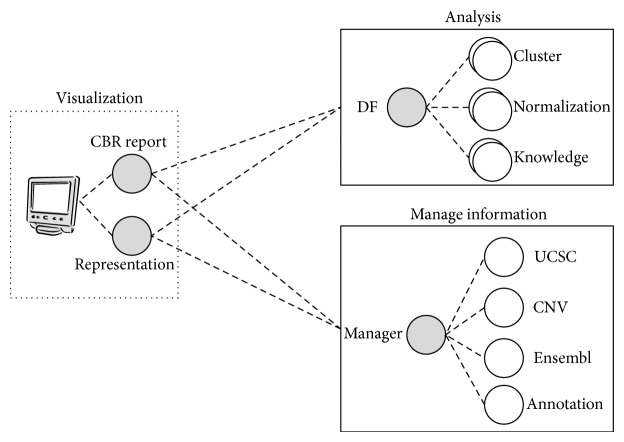
Multiagent system architecture.

The multiagent system is composed of three layers: analysis, information management, and visualization.  Figure 1 shows the multiagent system architecture and the layers it comprises. The analysis layer performs the microarray analysis. It includes several algorithms that can be applied to the specific case study taken into consideration. The information management layer generates a local database using the information of several sources. The visualization layer manages the information and the algorithms. It displays the information and the results obtained after applying the existing algorithms at the analysis layer.

The analysis layer contains the DF (Directory Facilitator) agent, which registers the different types of agents that are contained in that layer and the services they provide. Each agent provides a set of services to perform certain functionalities that can be requested by other agents. This separation allows the inclusion of new functionalities in the application by modifying the services provided by the agents at the analysis layer.

The information management layer contains an agent for each external source of information. Each agent is responsible for retrieving the information requested by the Manager agent, which compiles the information from the agents and generates a local database.

The agents at the visualization layer consult with the DF to obtain the services provided for each existing process. Additionally, these agents can contact the Manager in the information management layer to obtain relevant information that appears during the analysis.

The following subsections describe each one of the layers adapted to the case study for CGH arrays.

### 3.1. Analysis Layer

The agents in these layers perform different tasks for processing information, specifically the processing tasks that will be used in the case study and the chips. It is necessary to take into account that the algorithms can be adapted to each case study. In the particular case presented in this work, this layer contains agents for normalization and segmentation, knowledge extraction, and clustering.

#### 3.1.1. Normalization and Segmentation

During this process, the data are preprocessed in order to segment them and reduce noise. This state is important to represent the information and extract relevant regions. While there are many algorithms capable of carrying out the preprocessing, the package snapCGH [18] R Server was used in this tool to normalize and segment the data. The package incorporates algorithms as aCGH, DNACopy, and GLAD [26, 27]. It is important to use algorithms in order to compare the different results; additionally, this package is widely used.

In order to compare all of the arrays simultaneously, the value is readjusted for all data that were previously processed by NimbleGen, which normalizes and segments each of the arrays. The data are then scaled according to the mad1dr (median absolute deviation) provided by NimbleGen. The process is defined according to the function

(1)fx,m,tl,tgfax,m,tlx≤0fbx,m,tgx>0,(2)fax,m,tl=f1x,m,tlflx,m,tl<00flx,m,tl≥0,fbx,m,tl=fgx,m,tlfgx,m,tl<00fgx,m,tl≥0,(3)flx,m,tl=x−−tl−m−−m−x−tl−mm,fgx,m,tg=x+tg−m−m−xtg−mm,

where *x* represents the value of the segment, *m* is the value of the mad1dr for the given array, tl is the loss threshold, and tg is the gain threshold.

#### 3.1.2. Knowledge Extraction and Classifiers

There are currently several kinds of classifiers based on different technologies: decision rules and decision trees RIPPER [28], One-R [29], M5 [30], J48 [31], CART [1] (classification and regression trees), probabilistic models naive Bayes [32], fuzzy models K-NN (K-nearest neighbors) [33], neural networks [34], and so forth. Some of these classifiers can be used to extract relevant information in order to obtain attributes, and this process can be carried out by traditional statistical techniques to compare values using the parametric or not parametric test ANOVA [35], Kruskal-Wallis [36], and Mann-Whitney *U* test [37] or testing to compare the frequencies as parametric Chi squared [38] or Fisher's exact test. The gain functions are a particular case of the techniques used in decision trees and decision rules for selecting the attributes, which is why they are not considered separately.

For this particular system, the decision trees were chosen to select the main genes of the most important pathologies, specifically the J48 [31] in its implementation for Weka [39]. However, if the system needs a generic selection, gain functions or statistical test is chosen (specifically Chi squared).

Chi squared test [38] was selected because it can work with qualitative and nominal variables and it provides an easy way to select relevant regions depending on a *P* value. Fisher's exact test [40, 41] is applied, which is the recommended method when the sample size is small, and it is not possible to ensure that 80% of the expected frequency from a contingency table has a value greater than 5.  Table 1 displays the information used in the contingency tables to carry out the statistical tests.

The segments that were considered most relevant for each of the CGH arrays were selected for each pathology.  Algorithm 1 displays the selection algorithm for the relevant segments used for the set of arrays and for the individuals with or without a particular pathology, as identified by the groups variable. The algorithm was applied repeatedly for each existing pathology.

#### 3.1.3. Cluster

There is a wide range of possibilities in data mining. Some of these techniques are artificial neural networks such as SOM [42] (self-organizing map), GNG [43] (growing neural gas) resulting from the union of techniques CHL [44] (competitive Hebbian learning) and NG [45] (neural gas), and GCS [43] (growing cell structure). There are other techniques with fewer computational costs that provide efficient results. Among them we can find the dendrogram and the PAM method [46] (partitioning around medoids). A dendrogram [47] is an ascendant hierarchical method with a graphical representation that facilitates the interpretation of results and allows an easy way to establish groups without prior establishment. The PAM method requires selecting the number of clusters previous to its execution.

Dendrograms are hierarchical methods that initially define conglomerates for each available case. The algorithm is modified so that each coordinate stores the values −1, 0, and 1 to indicate that the segment has a loss, no variation, or a gain, respectively. At each stage, the method joins the two conglomerates with the least distance and then calculates the distance of the other conglomerate to this new one. The new distances are updated in the matrix of distances. The process finishes when there is only one conglomerate remaining (agglomerative method). The distance metric used in this paper was the average linkage, a metric that calculates the average distance of each pair of nodes for the two groups and, based on these distances, merges the groups. The metric is known as the unweighted pair group method using arithmetic averages (UPGMA) [48]. This type of cluster was selected since it allows the grouping process to be easily reviewed by visualizing the results. Dendrogram algorithm is described in Algorithm 2.

### 3.2. Information Management

The information management layer includes a different agent for each available source of information that is managed by the Manager agent. The specific agents used are UCSC, Ensembl, CNVs (copy-number variations), and annotation [49]. These agents download existing information from the databases managed by the Manager agent to generate the local database. They specifically download information related to genes, proteins, pathologies, genomic variants, and CNV. This information is compiled by the Manager agent, who is responsible for generating a local database that will be used as a source of information. In addition to the information retrieved from the database, the system stores the annotations created by the system experts for future data analysis.

**Figure 2:**
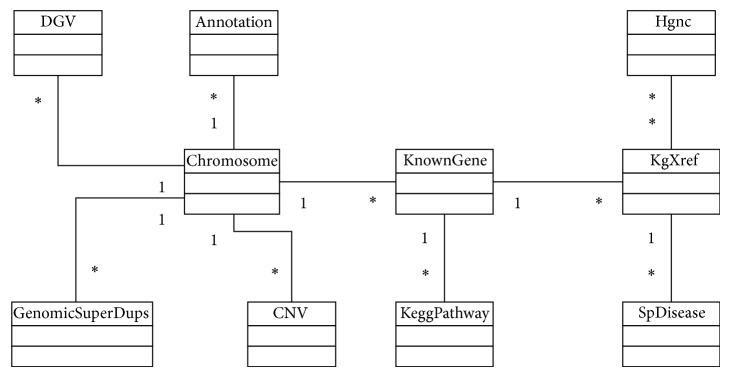
Class diagram with the information stored in the databases.

When the information in the GUI database requires updating, the Manager agent orders the agents to download the updated information from the remote websites. Using this information, the agent then stores the data in the system's local database in order to improve performance. There are different local databases for the different versions of HG that are being used. The data model used for each of the databases follows the class model shown in Figure 2.

Although the information from the tables is downloaded from UCSC, the data model does not follow the same diagram; the information stored in the tables does, however, correspond to the information that can be found for the equivalent tables in UCSC:

- DGV: database of genomic variants,
- annotation: comments that are inserted into regions of the chromosomes,
- chromosome: table with information for the chromosomes which only stores the chromosome identifier,
- CNV: table that stores information used to represent the copy-number variations,
- KnownGene: table with information about the genes,
- KeggPathway: KEGG pathway cross-reference,
- KgXref: linking together a known gene ID and a gene alias, used to extract the information commonly used to identify genes,
- Hgnc: a cross-reference table between HUGO Gene Nomenclature Committee (HGNC) IDs and other database IDs,
- SpDisease: a cross-reference table between Swiss-Prot IDs and disease description.

The advantage is that all of the information is generated in a single database and stored locally, which improves performance; additionally, new tables such as CNV can be added, or further annotations can be provided to the database.

### 3.3. Visualization

aCGH is a technique to detect variations in patients who have different mutations in chromosomal regions. Usually the variations have already been catalogued, which is why the existing information can be used to catalogue and evaluate the mutation. In this case study, the cases were defined according to the segments in which the chromosomic regions have been fragmented. Therefore, in a CBR system [50], the retrieval and selection phase is adapted to get the most suitable information that solves the problem.

**Figure 3:**
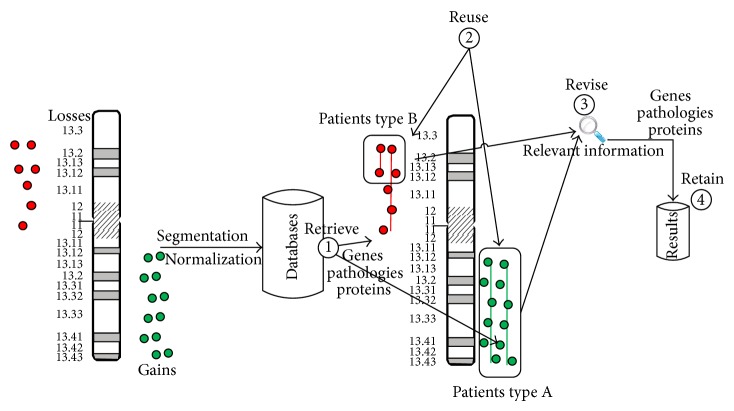
Chromosome 19 losses in red and gains in green.

Cases involving stored memory correspond to the information for the region and the information associated with the region. There are cases associated with genes, pathologies, CNVs, annotations, variants, and duplications. The algorithm selected for the retrieval of cases should be able to search the case base and select the genes, the known transcripts associated with the region, the variations to gains or losses, and so forth in the regions. The retrieved genes and transcripts are shown with each of the segments to validate the obtained results using the analysis techniques. The revision phase is carried out by an expert, and finally the retained phase allows storing the information considered relevant. The analysis process followed by the system is shown in Figure 3.

During the retrieval stage, the information previously stored by the Manager agent is retrieved from databases such as UCSC or Ensembl. The retrieved information is that which is considered the most relevant and is organized in order to generate local databases, which are completed with other existing information, such as that originating from the copy-number variations.

The stored information is reused during the reuse phase in order to generate the reports, which are provided to the end user, on the analysis of the regions that stand out during a visual analysis or automatic analysis of the data. Part of this information is also used to draw regions and nonrelevant mutations, which helps the subsequent revision of the selected segments as relevant.

The revision phase is carried out by an expert who determines the relevance of the selected regions according to the variations and the information retrieved from the reports. The expert also inputs any annotations considered relevant regarding the detected variations, which are stored during the learning phase for future analysis of new patients. Moreover, the revision is facilitated by representing information such as the CNVs and the annotations, which eliminates the variations that are not considered relevant.

## 4. Results

The multiagent system designed in this work was applied to the study of CGH arrays. A functionality was developed to study the different types of arrays. The system was applied on three different kinds of CGH arrays: BAC aCGH, oligo aCGH, and SNP CGH. Although these arrays are similar, the information provided by each differs considerably because the segments are defined in different ways.

The first step in the analysis of CGH arrays is the segmentation and normalization process; Table 2 shows the information obtained from the BAC arrays after this step. In this kind of array, all patients are represented by the same segments, shown as rows in the image. Each segment contains information about the chromosome, initial position and final position. For each region, we have a value *v*_*ij*_ with the information of gains and losses for the segment *i* and patient *j*; these values represent gains or losses if they are greater or lower than a threshold.

An analysis using oligo aCGH shows that the available information is different. The information from these arrays is shown in Table 3. The values *v*_*ij*_ represent gains and losses for segment *i* and patient *j*. Each patient has a different number of segments, which might not have the same initial value; this means that the initial or final value of segment *i* and patient *j* can be different from segment *i* and patient *k*.

Finally, the system includes databases since the system extracts the information regarding genes, transcripts, CNV, and local annotations.

**Figure 4:**
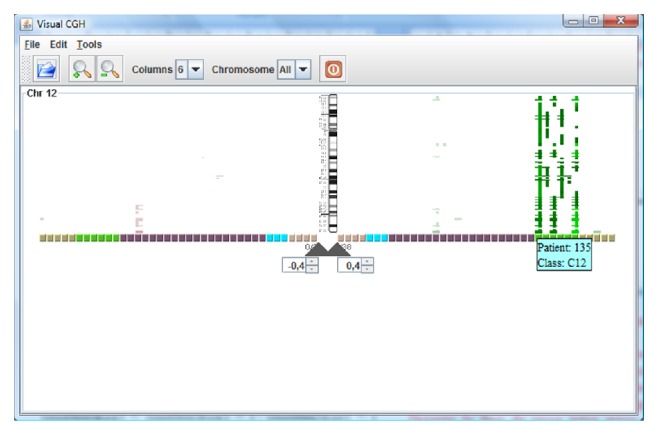
Automatic selection of segments.

Figure 4 shows the information from the BAC arrays cases, which includes 38 cases with 5 different pathologies. Only the information corresponding to chromosome 12 is shown. The green lines show gains regions in the chromosome, while red lines show losses. Therefore, the figure shows that the green patients have gains while the remainder presents few variations. The most relevant segments are automatically highlighted as bright segments with the application of the hypothesis contrast Chi squared test. This technique allows the selection of the regions of interest.

**Figure 5:**
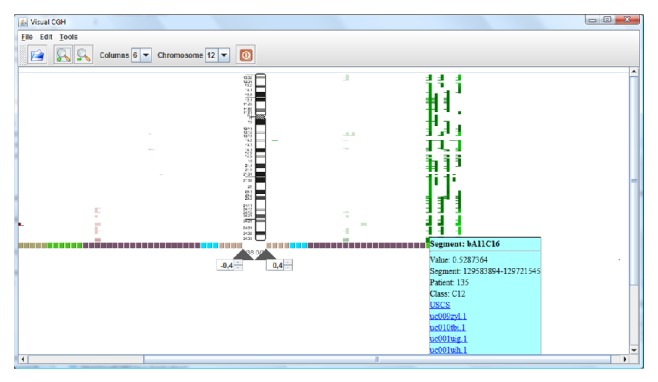
Automatic selection of segments and genes.

Once the data are represented, a CBR reasoning cycle is performed. During the retrieval phase, information regarding the catalogued genes and transcripts is recovered from the database. During the reuse phase, these genes are evaluated and valued according to the hypothesis contrast described in Section 3. After selecting the segments, their relevance can be observed.  Figure 5 shows the information from the genes that were recovered from the database and considered to be relevant.

**Figure 6:**
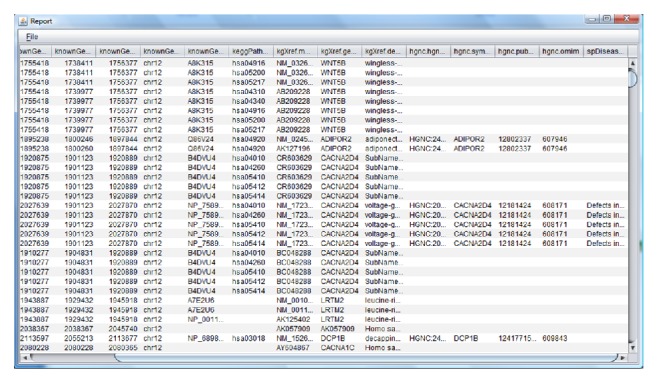
Automatic report with information from the highlighted segments.

In addition to visualizing the information for each of the different segments, it is also possible to generate reports automatically. These reports make it possible to quickly visualize potentially relevant information from the regions about proteins, genes, and diseases.  Figure 6 displays information related to proteins, genes, and diseases. The system also makes it possible to generate reports on variants, duplications, or CNVs.

**Figure 7:**
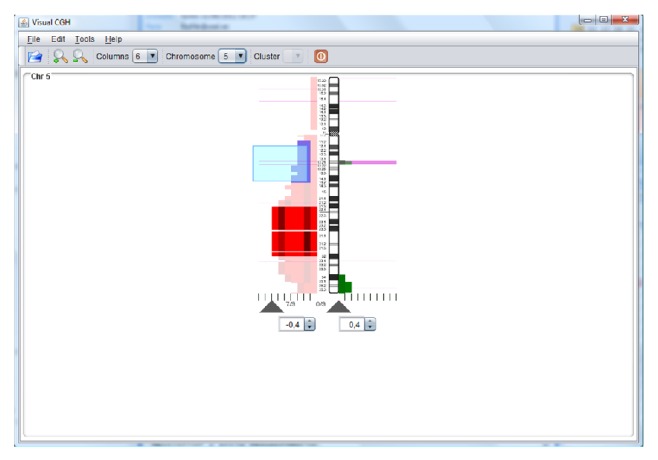
Accumulated view of chromosome 5 together with information from the CNVs.

The system provides several visualizations to support the revision of the information by an expert. The system allows for information about the previously analyzed regions to be included, which makes it possible to eliminate regions that were not previously considered relevant. Additionally, the system can include representations of different variations, which makes it possible to eliminate regions whose mutations have already been catalogued as not relevant. This helps the visual analysis and selection of relevant regions. The pink area highlighted in Figure 7 represents the regions presented by CNV; there is a gains area highlighted in green which corresponds to a CNV and should not be taken into consideration in the analysis. The information from the annotations inserted by laboratory personnel is likewise shown, with the color varying according to the user's selection. Another variation with respect to Figure 4 is that the data are represented as an accumulated amount as opposed to per individual patient, which allows the regions of gains or losses to be easily observed. The regions that were automatically selected by the analysis tests can be modified by using a mouse to mark the segments with a selection square or by dragging the triangles located in the lower part of the chromosome.

**Figure 8:**
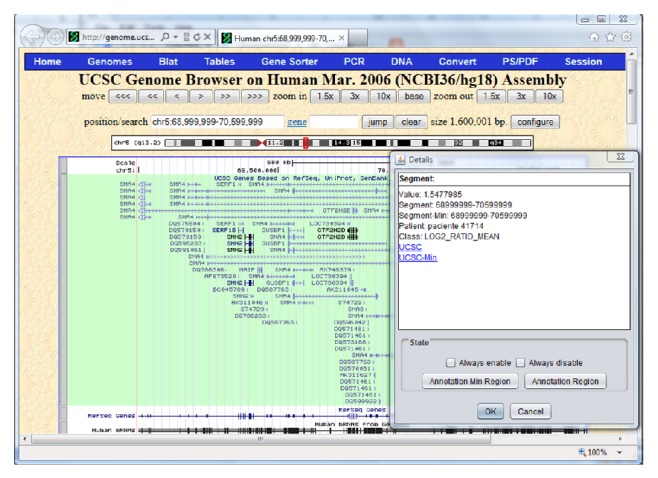
Information from UCSC for the selected segment.

If there are any doubts, it is possible to consult the UCSC website to determine the relevance of a specific segment; to see the accumulated view, simply click on the segment and then select OK for the UCSC option for either the complete segment of the patient or the minimum region into which each segment has been resegmented.  Figure 8 shows the window from the UCSC site and the dialogue box that appears after clicking on a particular segment. This information is useful to analyze the relevance of the regions by taking into consideration the different knowledge extraction techniques applied.

**Figure 9:**
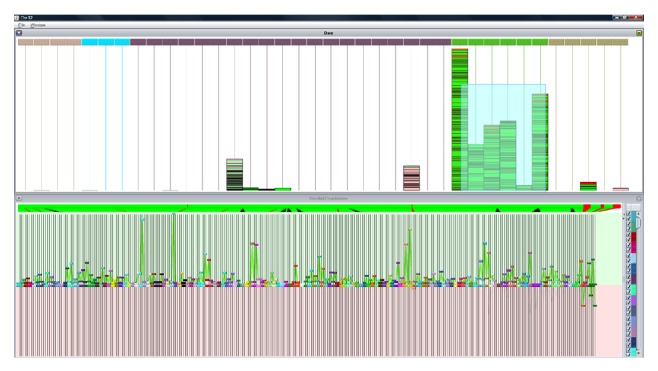
Clustering review with a bar graph and parallel coordinates.

Figure 9 shows a representation in parallel coordinates and a bar graph. In the bar graph, one bar represents each individual and is divided into different segments with an amplitude proportional to the width of the segment. The color of the top rectangle represents the type of pathology the patient has. In parallel coordinates, each line is associated with a patient and the color represents the pathology type. Each coordinate represents a segment. If we select the patients from the green category in the stacked bars, we can see how the other bars are deactivated, which indicates that the patients have variations within different ranges; only the patients with variations within the range of the selected patients remain active, which makes it easy to see other similar patients. In the parallel coordinates, the values of each coordinate are adjusted to the maximum and minimum extremes for the selected individuals. The lines for each selected individual are highlighted while those not within the range of maximum and minimum values as established for each coordinate are marked in gray.

In the case of Figure 9, the number of segments selected was very high, which explains the appearance of so many coordinates. Having selected fewer, the number of coordinates would be lower, making it easier to see the range of variation for each of the coordinates.

**Figure 10:**
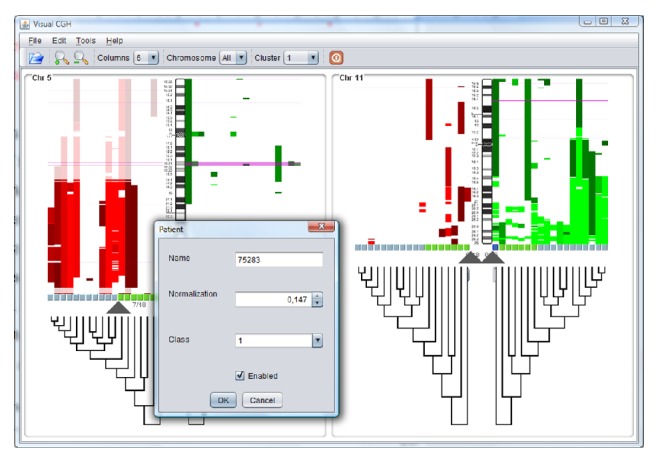
Grouped individuals according to the highlighted segments for each chromosome.

If there are no groups, the system can also create a cluster of individuals which can then be revised by selecting the individuals with a mouse and modifying the cluster to which they belong. Clusters can be made only according to the information from the chromosomes that can be seen on the screen and only based on the highlighted segments, which makes it possible to create a group according to the information considered relevant.  Figure 10 shows a cluster created from chromosomes 5 and 11 using all of the information from chromosome 11 and the highlighted information from chromosome 5. Once the dendrogram was created, the cluster was manually corrected by selecting individuals one by one.

## 5. Conclusions

Visualization also makes it possible to carry out tasks, such as drag and drop, for each visualization, to export information in image format, to select thresholds, chromosomes to visualize, categories, individuals, to zoom, or to import Affymetrix (multiple tsv files) and NimbleGen (multiple txt files) data.

The multiagent system can add agents that specialize in specific case studies and allows the reuse of functionalities for specific layers. Furthermore, the independence of the different modules in this kind of system allows for the easy inclusion of new techniques. This case study used the aCGH data analysis to facilitate the addition and/or modification of existing techniques. The system provides easy access to information of several databases, improving the visual analysis of the information and proving relevant information of the selected regions of the chromosome. The system uses CBR to automatically select the genes that characterize pathologies. This CBR manages all the information of the databases and it allows the incorporation of new information that can be used in future analyses.

Finally, the different visualization can easily manage the data, thus improving the efficiency of the experts in the selection of relevant regions, its validation, and the access to information associated with these regions.

## Figures and Tables

**Algorithm 1:**
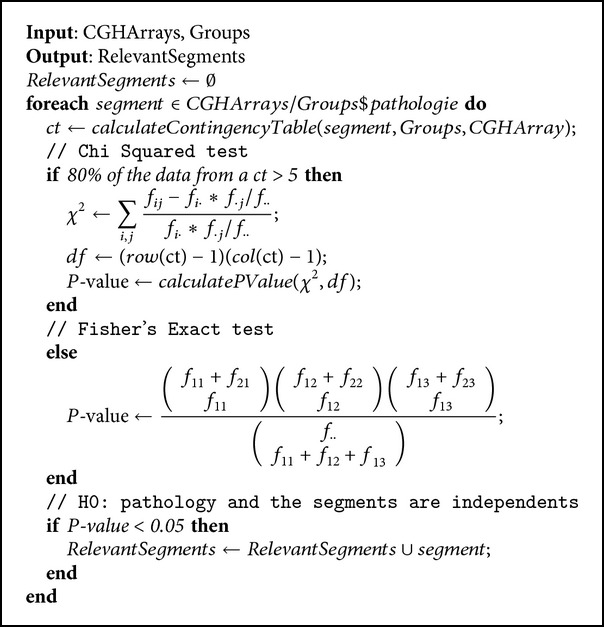
Automatic selection of segments.

**Algorithm 2:**
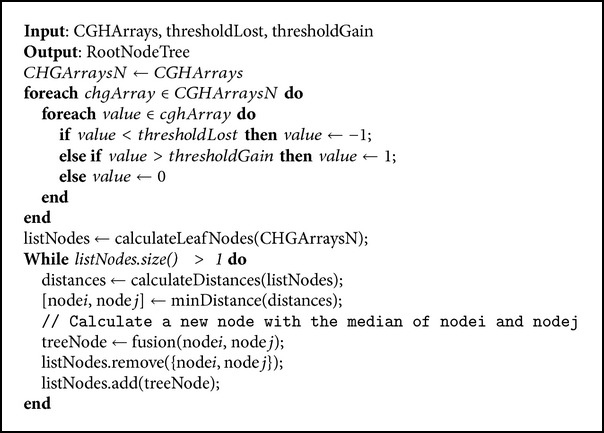
Dendrogram algorithm.

**Table 1:** Contingency table.

	Gain	Normal	Loss	Total
Pathology	*f*_11_	*f*_12_	*f*_13_	*f*_1·_ = total pathology
Not pathology	*f*_21_	*f*_22_	*f*_23_	*f*_2·_ = total not pathology

Total	*f*_·1_ = total gains	*f*_·2_ = total normal	*f*_·3_ = total losses	*f*_··_ = total

**Table 2:** BAC aCGH segmented and normalized.

	Patient 1	Patient 2	Patient 3	⋯	Patient *n*
Segment 1 (chromosome-init-end)	*V*_11_	*V*_12_	*V*_13_	⋯	*V*_1*n*_
Segment 2 (chromosome-init-end)	*V*_21_	*V*_22_	*V*_23_	⋯	*V*_2*n*_
⋮	⋮	⋮	⋮	⋮	⋮
Segment *m* (chromosome-init-end)	*V*_*m*1_	*V*_*m*2_	*V*_*m*3_	⋯	*V*_*mn*_

**Table 3:** Oligo aCGH segmented and normalized.

Patient 1		Patient 2		⋯	Patient *n*	
Segment 11 (chromosome-init-end)	*V*_11_	Segment 12 (chromosome-init-end)	*V*_12_	⋯	Segment 1*n* (chromosome-init-end)	*V*_1*n*_
Segment 21 (chromosome-init-end)	*V*_21_	Segment 22 (chromosome-init-end)	*V*_22_	⋯	Segment 2*n* (chromosome-init-end)	*V*_2*n*_
⋮	⋮	⋮	⋮	⋮	⋮	⋮
Segment *m*1 (chromosome-init-end)	*V*_*m*1_	Segment *m*2 (chromosome-init-end)	*V*_*m*2_	⋯	Segment *mn* (chromosome-init-end)	*V*_*mn*_
		⋮	⋮	⋮	⋮	⋮
		Segment *k*2 (chromosome-init-end)	*V*_*k*2_	⋯	Segment *ln* (chromosome-init-end)	*V*_*ln*_
